# Integrative Approaches in Structural Biology: A More Complete Picture from the Combination of Individual Techniques

**DOI:** 10.3390/biom9080370

**Published:** 2019-08-14

**Authors:** Linda Cerofolini, Marco Fragai, Enrico Ravera, Christoph A. Diebolder, Ludovic Renault, Vito Calderone

**Affiliations:** 1 Center for Magnetic Resonance (CERM), University of Florence, Via L. Sacconi 6, 50019 Sesto Fiorentino (FI), Italy; 2 Department of Chemistry ‘Ugo Schiff’, University of Florence, Via della Lastruccia 3, 50019 Sesto Fiorentino (FI), Italy; 3 NeCEN, Institute of Biology Leiden, Leiden University, Gorlaeus Gebouw, Einsteinweg 55, 2333 CC Leiden, The Netherlands

**Keywords:** integrative structural biology, X-ray crystallography, solution NMR, SSNMR, SAXS, cryo-EM

## Abstract

With the recent technological and computational advancements, structural biology has begun to tackle more and more difficult questions, including complex biochemical pathways and transient interactions among macromolecules. This has demonstrated that, to approach the complexity of biology, one single technique is largely insufficient and unable to yield thorough answers, whereas integrated approaches have been more and more adopted with successful results. Traditional structural techniques (X-ray crystallography and Nuclear Magnetic Resonance (NMR)) and the emerging ones (cryo-electron microscopy (cryo-EM), Small Angle X-ray Scattering (SAXS)), together with molecular modeling, have pros and cons which very nicely complement one another. In this review, three examples of synergistic approaches chosen from our previous research will be revisited. The first shows how the joint use of both solution and solid-state NMR (SSNMR), X-ray crystallography, and cryo-EM is crucial to elucidate the structure of polyethylene glycol (PEG)ylated asparaginase, which would not be obtainable through any of the techniques taken alone. The second deals with the integrated use of NMR, X-ray crystallography, and SAXS in order to elucidate the catalytic mechanism of an enzyme that is based on the flexibility of the enzyme itself. The third one shows how it is possible to put together experimental data from X-ray crystallography and NMR restraints in order to refine a protein model in order to obtain a structure which simultaneously satisfies both experimental datasets and is therefore closer to the ‘real structure’.

## 1. The Importance of Integrated Structural Biology

The elucidation of the structural features of macromolecules by X-ray and NMR has had a great impact on the research in chemistry, biology, and medicine. Today, the scientific and technical progress has allowed for expanding the capability for structural biology to investigate systems not accessible, so far, to conventional methodologies and to characterize the dynamic processes and transient interactions both inside and outside the cell [1,2,3,4,5,6,7,8].

Problems concerning crystallization [9], the size of the macromolecule [10,11] to study, and the heterogeneity of the sample are no more obstacles to the structural characterization. X-ray diffraction and NMR are still the largest contributors to the field, but electron microscopy is gathering pace. At the same time, NMR spectroscopy has also undergone technical and methodological advancements now allowing for the characterization of difficult systems such as very large or transient protein complexes, fibrils, and protein embedded in matrices [12,13,14,15].

However, the complexity of the biological systems is such that none of these methodologies alone can provide a full picture of the structural, dynamical, and functional properties that are of relevance to biology [16,17]. The integration of the different biophysical methodologies with the support of the computational methods is thus clearly the strategy of choice to deal with the most interesting biological macromolecules and pathways [18]. This approach has proven to be efficient also in the characterization of synthetic and semi-synthetic macromolecules designed as drugs or drug candidates and to investigate processes where macromolecules rapidly sample multiple conformations. In other cases, the simple knowledge of the three-dimensional structure of a biomolecule is not enough for understanding the mechanism of the biological process in which the biomolecule is involved.

We will here revisit three cases taken from some recent research in our laboratory that allow to further stress the importance and the power of such an integrated approach.

### 1.1. The Characterization of polyethylene glycol (PEG)ylated l-Asparaginase Suggests a Strategy for the Investigation of ‘Coated Biologics’

One of the major targets in nanomedicine is the development of nanocarriers to deliver drugs to specified locations. Despite in vitro experiments carried out using the drug-loaded nanocarriers being successful, the efficiency of these drug carriers in vivo is not encouraging as they fail to reach their target. This efficiency depends on the route of administration, on the preferential uptake by non-target cells, on the lifetime of the carrier within the biological system, and on the ability of the nanocarrier to escape the immune system response to its presence. The number of approved biological drugs against several diseases [19,20] is increasing. Some of them have been investigated by X-ray crystallography [21,22,23], NMR spectroscopy [24], and, more recently, by cryo-electron microscopy (Cryo-EM) [25] to obtain structural information potentially useful in their optimization by rational design.

For this reason, the characterization of “stealth biologics”, which are able to efficiently reach their target, is an attractive area of research, is becoming a must for the approval of new biologics by the authorities [26,27,28,29], and is an illuminating example of the great opportunities provided by the integration of the different biophysical methodologies. Example “stealth biologics” are cytokines, enzymes, and growth factors conjugated with chains of polyethylene glycol (PEG) to improve the pharmacokinetic properties and lower the immunogenicity. In particular, the coating of proteins with PEG is used to produce “stealth biologics” largely invisible to the immune system, stable to the enzymatic hydrolysis, and with a reduced renal clearance.

However, PEGylation is a challenge for structural biologist: (1) It inhibits the crystallization of the protein [30], (2) causes serious problems to the standard structural biology techniques [31,32,33,34,35], and (3) limits the use of solution NMR to the small proteins because it significantly increases the hydrodynamic volume of the protein itself [30,36,37,38,39]. Therefore, it is not possible to obtain information at the atomic level to drive any possible structure-based optimization processes using only a single biophysical method.

Recently, we have shown that the integration of NMR and X-ray crystallography allows for the obtainment of an experimental and reliable structure of a large PEGylated protein [33].

Our group has recently reported high quality solid-state NMR spectra (SSNMR) of some PEGylated proteins [37]. The comparison of ^13^C-^13^C two-dimensional SSNMR spectra collected on some native proteins and on their PEGylated forms proves that in all cases 3-D structure of the protein core is preserved after conjugation with PEG [37]. One of these, l-asparaginase II (ANSII), is currently used as native enzyme or in its PEGylated form to induce l-asparagine depletion in patients with acute leukemia [40,41]. The tetrameric structure of the native enzyme was revealed by X-ray crystallography. The four identical monomers (326 amino acids) are organized to form a dimer of dimers of 138 kDa with a D2 symmetry [23]. The conjugation of the exposed lysines with short PEG chains (of about 1 kDa) does not allow the collection of NMR spectra in solution on the tetrameric protein. Conversely, the presence of the PEG chains does not prevent the collection of SSNMR spectra on rehydrated sample of the protein to perform resonance assignment and to obtain structural restraints.

Taking into account the complexity of the problem, experimental data obtained from different biophysical methodologies were analyzed following an integrated approach to properly characterize this PEGylated protein [2,42].

To characterize these large PEGylated proteins/complexes, we relied upon a combined use of solution NMR and X-ray crystallography and solid-state NMR experiment collected on the conjugated l-asparaginase. Here, the previous analysis was complemented with cryo-EM data to further validate the structural models calculated by the experimental restraints.

**Scheme 1:**
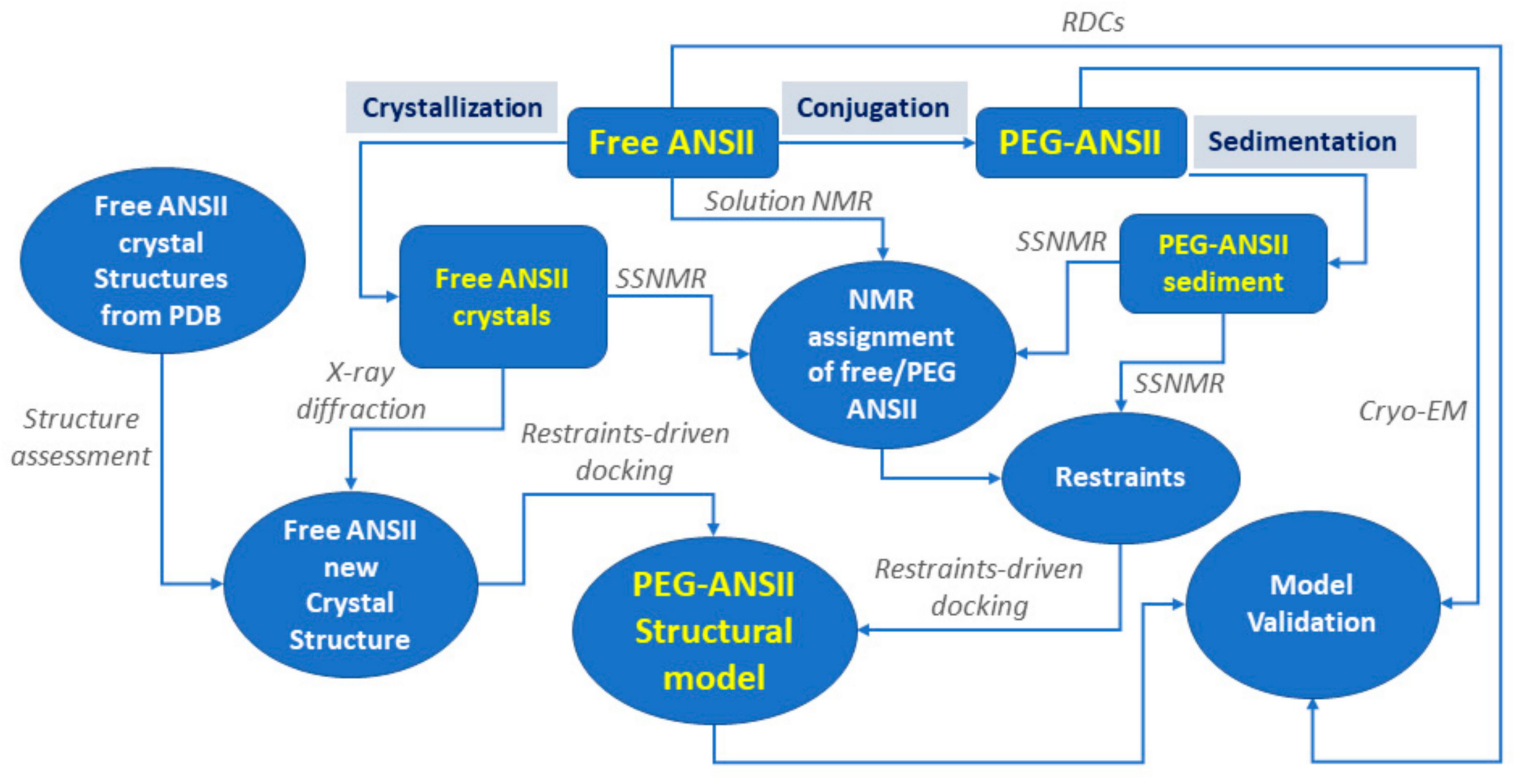
Schematic representation of the integrated approach used for the structural characterization of polyethylene glycol (PEG)ylated l-asparaginase II (ANSII).

The used strategy has been the following: (i) 2D SSNMR spectra was recorded on the same batch of crystals used to solve the X-ray structure of l-asparaginase II and on rehydrated samples of the PEGylated protein in the form of a sediment; (ii) the two sets of SSNMR spectra are compared for a first structural assessment; (iii) solution NMR spectra on a deuterated sample of l-asparaginase II was collected to assign the protein regions retaining a large mobility; (iv) 2D and 3D SSNMR spectra were collected on crystalline and PEGylated samples of the protein to complete an extensive resonance assignment, integrating the data collected in solution; (v) structural restraints are derived from ^13^C-^13^C two-dimensional SSNMR spectra to confirm the fold of both the native protein in solution and of its PEGylated form; (vi) a restraints-driven docking calculation is used to generate a representative structural model that is compared to 2D classes from cryo-EM and validated using the residual dipolar couplings (RDCs) collected in solution using a filamentous phages Pf1 as alignment medium on the free protein (Scheme 1).

PEGylation of ANSII does not appear to alter the overall architecture of the protein, as shown in the Cryo-EM 2D classes derived from native as well as PEGylated ANSII particles (see Figure S1). Both sets of 2D classes show particles of around 9 nm in length and 7 nm in width, compatible with the size of the ANSII tetramer. Interestingly, the presence of PEG does not affect the Cryo-EM determination of the overall protein shape.

**Figure 1:**
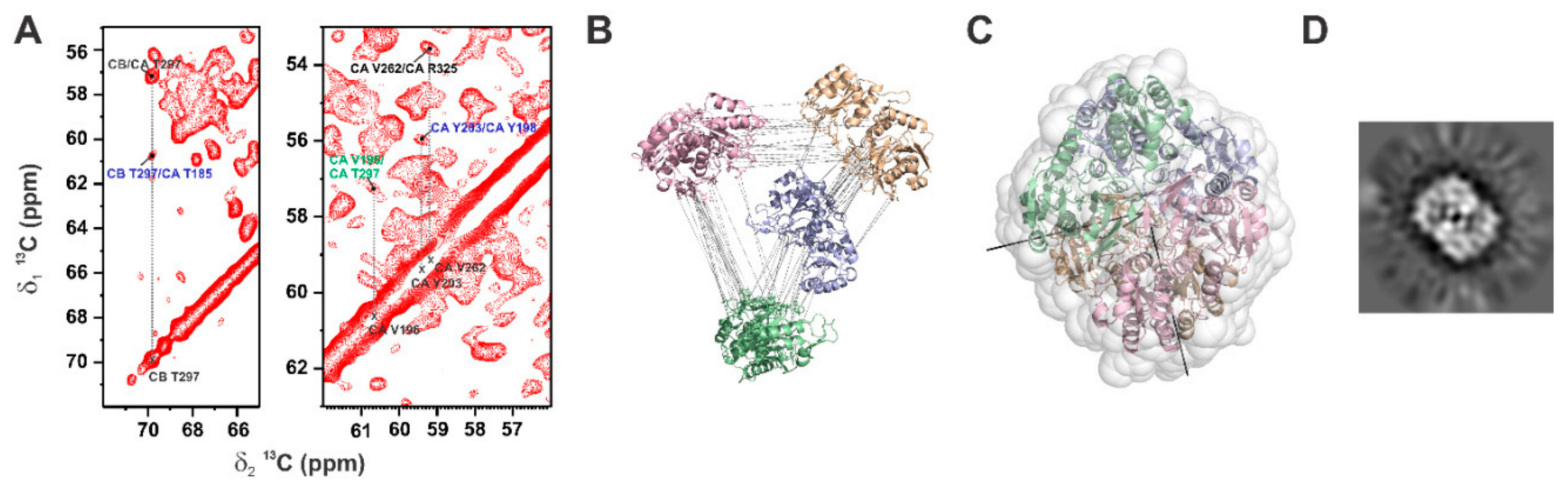
(**A**) Enlargement of two regions of the 2D ^13^C-^13^C DARR (Dipolar Assisted Rotational Resonance) solid-state NMR (SSNMR) spectrum (mixing time 200 ms) displaying long-range inter-residue restraints (blue interdimer and green interdomain/intramonomer). (**B**) Representation of the 21 intermonomer distance restraints used in HADDOCK calculations. Ten of these restraints define the interface between the two monomers (chains A and C in wheat and pink, respectively) forming the intimate dimer, whereas eleven define the interface between other two monomers (chains A and D in wheat and light-blue, respectively). The restraints were replicated according to the protein symmetry. (**C**) Structural model with the lowest energy obtained with HADDOCK calculations, implementing SSNMR experimental restraints and residual dipolar couplings (RDCs) restraints. The PEG chains are schematized as white spheres. (**D**) Cryo-electron microscopy (cryo-EM) 2D classes of ANSII PEGylated with PEG 5000. The figure is an adaptation from Reference [21].

Collectively, NMR and Cryo-EM data demonstrate that the PEGylated ANSII retains the quaternary structure of the native enzymes. Moreover, structural model with the lowest haddock-score calculated starting from the restraints derived from the SSNMR spectra agrees with the RDCs collected on the native protein in solution (Table S1 and Figure S2A). For this reason, RDCs were added directly in the HADDOCK calculation in order to obtain a more accurate [43,44] structural model of PEG-ANSII in better agreement with all the collected experimental data (Figure 1). The calculation was repeated using inter-monomer distance restraints from SSNMR for the PEGylated protein together with the RDCs evaluated in solution on the free protein. Models obtained by including RDCs in HADDOCK calculations showed improved statistics compared to the models obtained using solely the SSNMR restraints (see Table S1, Table S2 and Figure S2). Furthermore, the calculated model is in agreement with the Cryo-EM 2D classes.

To date, there is still an open debate on the preservation of the protein structure after conjugation with large polymers. The use of this integrated strategy extends the possibility of structural biology to characterize these challenging systems with potential benefits for molecular pharmacology.

### 1.2. Integrated Analysis of the Molecular Mechanism of Collagen Proteolysis by Matrix Metalloproteinase-1 (MMP-1)

Several enzymes are multidomain proteins which carry out their catalytic activity by sampling multiple conformations. The identification of the conformations adopted by the enzyme in solution is required to describe in detail the catalytic mechanism. One significant example is represented by MMP-1 which, in its active form, is constituted by a catalytic and a hemopexin-like domain connected by a flexible linker. The isolated catalytic domain retains its hydrolytic activity against non-structured peptides but the degradation of highly structured substrates (i.e., collagen type I) requires the presence of the hemopexin-like domain.

In this example, we show that the integration of experimental restraints and computational methods can provide hints on the predominant conformations sampled by proteins in solution. Matrix metalloproteinases (MMPs) are a family of enzymes designed by nature to hydrolyze a variety of large proteins of the extracellular matrix [45,46]. MMPs have been evolved towards a broad proteolytic activity. At the same time the presence and activity of the enzyme are strictly regulated. As a result, the recognition and hydrolysis of a variety of substrates can be obtained by the interplay between high specialization of protein domains and protein interdomain flexibility [47]. The majority of MMPs’ active forms, including MMP-1, encompass two-domains, a catalytic (CAT) and a hemopexin-like (HPX) one, which can hydrolyze highly complex substrates, such as the triple-helical, interstitial (types I-III) collagen [48]. The substrate is much larger in size than the enzyme active cleft. Therefore, interdomain flexibility plays a crucial role in the catalytic mechanism: Interdomain rearrangements allows both the movement of the MMP along collagen fibrils and the untwisting/perturbation of the collagen helix, which is required for the positioning of a single peptide chain into the active site [47,49,50,51,52,53,54,55]. An accurate estimate of the most likely conformations in solution by the multidomain enzyme is thus required to explain the basis of substrate recognition and collagen hydrolysis at the molecular level. Experimental paramagnetic NMR spectroscopy and small angle X-ray scattering (SAXS) have been thus exploited to calculate the maximum occurrence (MO) of MMP-1 conformations (vide infra).

When a system is able to sample multiple conformations on the timescale of the experiment, the experimental data correspond to the weighted average of the “experimental observable” that would be observed for each conformation if the system was only sampling that specific conformation. To calculate the ensemble that is giving rise to a particular averaged observable is an “ill-posed inverse problem” that permits a number of plausible solutions [56,57]. A vast collection of methods, ranging from the identification of the sparsest ensembles to the maximum entropy reweighting [56,57,58,59,60,61,62,63,64,65,66,67,68,69,70,71,72,73,74,75,76,77,78,79,80,81,82,83,84,85,86,87,88,89,90,91,92,93,94,95] have been proposed to generate ensembles in agreement with the experimental data. Among those, in our laboratory a new method was developed aimed at identifying which are the conformations that are more likely accessed by the system. The method is called maximum allowed probability or Maximum Occurrence [80,96,97,98] and has the advantage of providing a mathematically correct upper bound to the population of a given conformation: The Maximum Occurrence (MaxOcc) of a given conformation or regions of conformations is defined and computed as the maximum weight that this conformation/region can acquire in any suitable ensemble while still preserving the ensemble’s ability to reproduce the experimental data. The MaxOcc approach was preferred over Maximum Entropy because of the unavailability of extensive MD trajectories to be reweighted.

**Figure 2:**
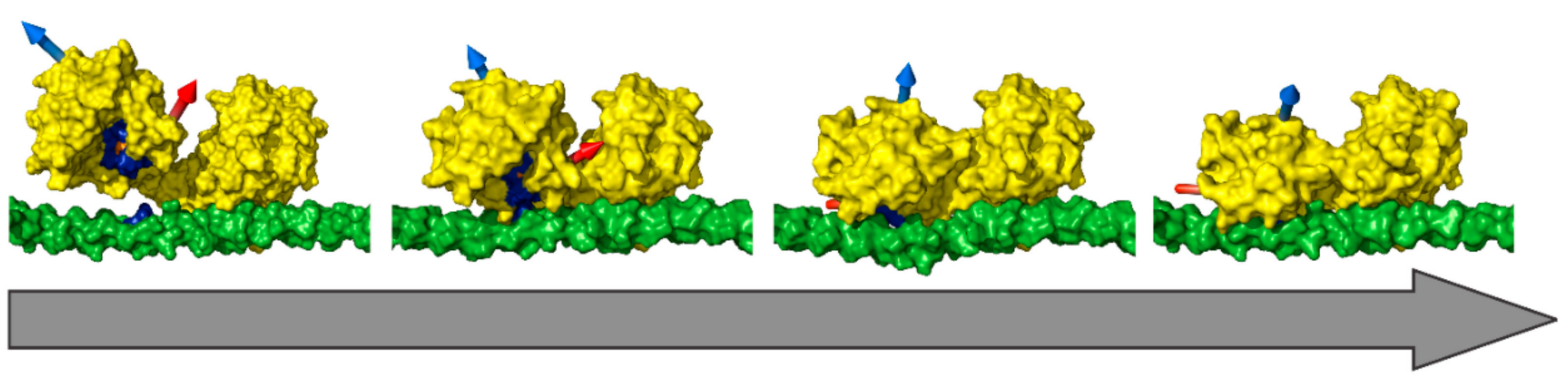
Modeling of the steps preceding the mechanism of collagenolysis. From left to right, one of the structures with the highest Maximum Occurrence (MaxOcc), two morphing intermediates, and the structure of the first step of the mechanism of collagenolysis. In yellow is the surface representation of matrix metalloproteinase-1 (MMP-1), in blue is the MMP consensus sequence HEXXHXXGXXH, in orange is the MMP-1 catalytic Zn^2+^, in green is the surface of a triple helical peptide model (THP) representative of collagen type I, and in blue is the THP cleavage site (Gly-Ile) of the first chain. The blue and red arrows indicate the directions of helices hA and hC, respectively, to facilitate visualizing the movement of the catalytic (CAT) domain with respect to the hemopexin-like (HPX) domain and the THP. The THP sequence is (GPO)4-GPQGIAGQRGVVGLO-(GPO)4 (where O is 4-hydroxyproline), based on the human α1(I) collagen chain. Adapted from Reference [100].

The MMP-1 conformations showing large MaxOcc values (up to 47%) are restricted into a relatively small structural region and are greatly different from the closed MMP-1 structures determined by single crystal X-ray crystallography. The MaxOcc of the latter is around 20%, which is somehow the upper limit for the existence of this conformation in the ensemble sampled by the protein in solution. In all the high MaxOcc conformations, the catalytic and hemopexin domains are not in close contact. Furthermore, the residues of the HPX domain that are responsible for the binding to the collagen are solvent exposed and ready to interact with the substrate. The MaxOcc analysis indicates that MMP-1 in solution is supposed to establish interactions with collagen and then to proceed steadily through the steps of collagenolysis (Figure 2) [99].

### 1.3. Integrating X-Ray and NMR Experimental Data

It is well known that combining different structural biology techniques can compensate for each other’s weaknesses or limitations. While “you cannot teach the eyes to hear or the ears to see” a more complete picture, which not only explains all the data but has also the possibility of highlighting hidden discrepancies, is obtained by the combination of different techniques [16].

NMR data, and in particular residual dipolar couplings, are precisely sensitive to the local features. The structural information about the overall shape of the molecule becomes accessible only if an increasing number of short-range data are included and with prior information about geometries. X-ray reflections encode primarily the information about the overall shape and can access the atomic details only when the number of high quality reflections at high resolutions increases. However, even at the highest resolutions, X-ray diffraction remains intrinsically poorly sensitive to local details.

NMR and X-ray diffraction have been presented historically in contrast to one another. Indeed, it is often the case that crystal structures and NMR data are not in agreement with one another. However, it has to be stressed that not in every case discrepancy is significant. This originates from the connate inaccuracy on short-range distances of X-ray crystallography, mainly when it comes to the determination of the position of hydrogen atoms, which are, on the contrary, the bulk of NMR experimental data. A very remarkable paper by Zweckstetter and Bax suggested that the inconsistency should be somehow “weighted” based on the X-ray data resolution and thus coined the term “structural noise” [100]. On passing, we note that the concepts reported in reference [100] had been dramatically overlooked by the NMR community, and this contributed to create the myth that it is most often the case that X-ray and NMR do not to agree. On the contrary, joint X-ray/NMR refinement has shown to be a very efficient way to reveal if discrepancies are real or merely due to structural noise. Recently, we have implemented the use of NMR restraints [101] into one of the most popular crystallographic refinement software, i.e., REFMAC [102,103] (which has been used to refine 42.7% of all the X-ray depositions in the PDB as of August 2019), and we have applied it to a number of examples [4,101,104].

**Figure 3:**
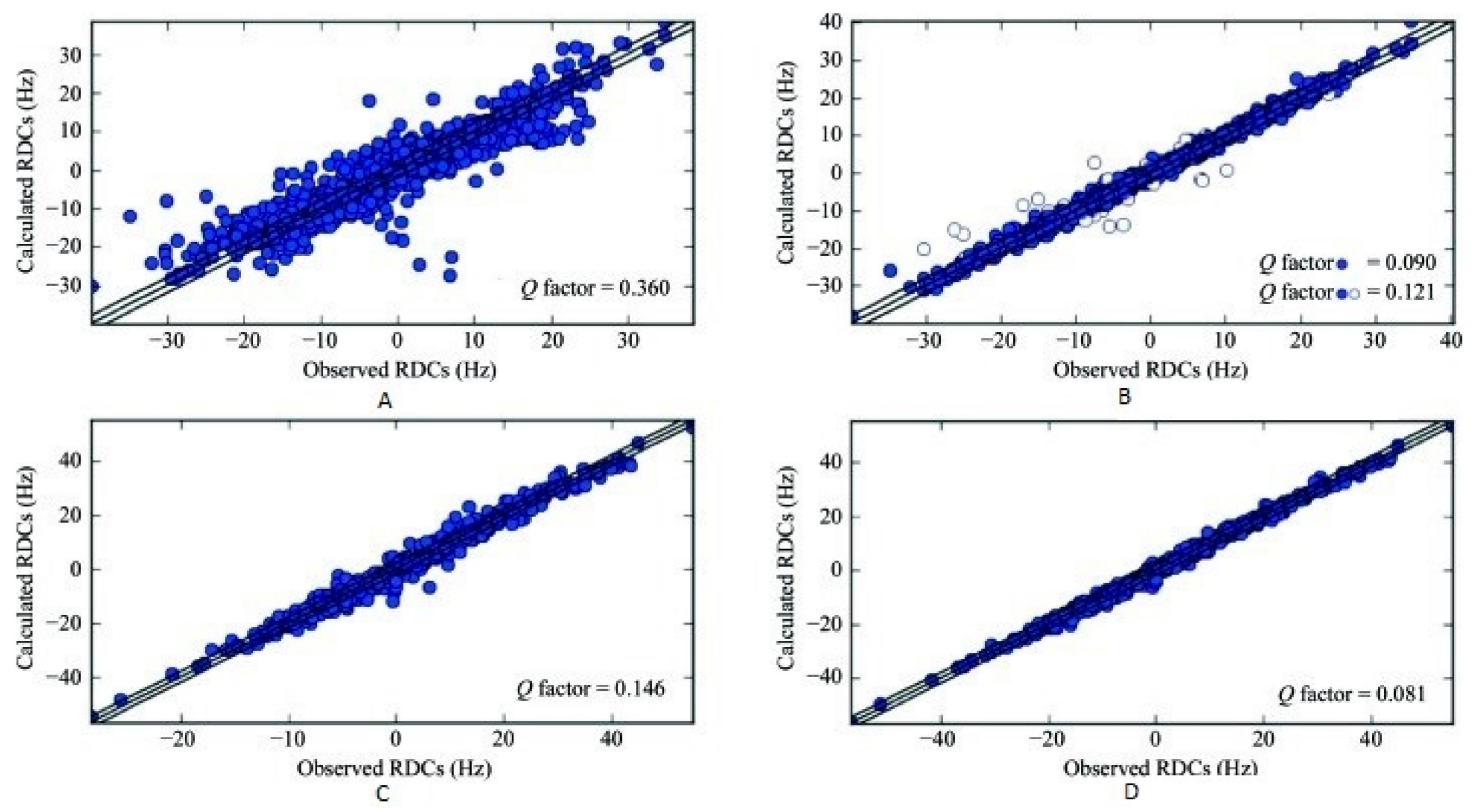
Calculated versus observed RDCs for ubiquitin (**A**,**B**) and GB3 (**C**,**D**) before (**A**,**C**) and after (**B**,**D**) joint refinement against RDCs and X-ray data. The empty symbols in (**B**) show the values for residues 8 and 72. Reproduced with permission from Ref. [101]

We here report the results of REFMAC-NMR calculations performed on two proteins that are extremely well characterized by NMR spectroscopy: Ubiquitin and GB3 (Figure 3). Especially in the case of ubiquitin, the apparent discrepancy between the X-ray structure and the NMR data (Figure 3, panel a) have been attributed to protein mobility [63,66]. However, a less strict interpretation of the X-ray structure demonstrated that the NMR data are largely compatible with a very limited mobility [105], and REFMAC-NMR clearly confirms this observation [101].

REFMAC-NMR was further modified so as to include a-priori knowledge of the specific system properties with the aim of decreasing the amount of data needed to achieve a more comprehensive scenario. In order to reinforce the efficiency of NMR for refining a multisubunit or multidomain system, it is possible to introduce constraints among the tensors used to fit the experimental data of the different domains or subunits. Differences in tensors can be due either to a rearrangement between the X-ray and the NMR structure or to the fact that different samples with different labeling patterns are used to minimize the complexity of NMR spectra. This allows for reducing the number of unknowns to which to fit the experimental data. It also allows for the improvement of the quality of refinements even when the experimental data cannot guarantee to accurately measure all the parameters, especially in those cases in which sizeable interdomain rearrangement is observed between the solution and the crystal [101].

The use of REFMAC-NMR shows that quite often NMR and X-ray data can be brought together to a single structure without calling in mobility issues [101].

We can conclude that joint refinement results in more accurate structures. In addition, it yields structural features that are in agreement both with the solid state and the solution state of the same molecule. This feature may be precious, for instance, when using the resulting structure as a starting point for molecular simulations or docking studies.

## Supplementary Materials

The following are available online at https://www.mdpi.com/2218-273X/9/8/370/s1, Figure S1: Cryo-EM micrograph and 2D classes of free ANSII (right) and ANSII PEGylated with PEG 5000 (left), Figure S2: Fit of the experimental RDC values collected in the presence of filamentous phages Pf1 (used as alignment medium) to the structural model with the lowest haddock-score calculated implementing either (A) only experimental SSNMR restraints (Qfactor 0.29), or (B) experimental SSNMR restraints together with RDCs (Qfactor 0.14) in HADDOCK 2.2, Table S1: Tensor parameters of the RDCs collected in the presence of filamentous phages Pf1 to the calculated structural models with the lowest haddock-scores. Table S2: HADDOCK statistics evaluated on the 200 water refined models of ANSII implementing either only experimental SSNMR restraints or experimental SSNMR restraints together with RDCs. The reported data are related to the best four structural models of the two main clusters with the lowest HADDOCK-scores.
