# Supplementary material for: Integrative Approaches in Structural Biology: A More Complete Picture from the Combination of Individual Techniques
Source: Biomolecules. 2019 Aug 14;9(8):370. doi: 10.3390/biom9080370 (PMC6723403; doi:10.3390/biom9080370)
Supplement: Supplementary file 1 [file biomolecules-09-00370-s001.pdf]

Supplemental methods

CryoEM of Native ANSII and PEG5000-ANSII

Sample preparation - CryoEM samples were prepared by plunge freezing after diluting concentrated ANSII and PEG-5000-ANSII in TRIS buffer (50 mM TRIS-HCl (pH 8), 10 mM $MgCl_2$). 4 μm ANSII (0.3 mg/mL) or PEG5000-ANSII (0.4 mg/mL) were applied to EM grids (Quantifoil R2/1) that have been glow discharged for 1 min at 30 mA (EMSK100X). Samples were plunge frozen in liquid ethane after blotting for 2 s using a robotic grid plunger (FEI VItrobot Mark IV). CryoEM data collection and processing are described in the supplementary information section.

CryoEM Data Collection and Processing of Native ANSII and PEG5000-ANSII

Data collection - Particles were imaged on a cs corrected FEI Titan Krios TEM at NeCEN. The microscope was operated at 300 keV and images were taken with a Falcon III direct electron detector, operated in linear movie mode with a pixel size of 1.44 Å and a total cumulative dose of 60 e-/A^2. In total, 291 micrographs of ANSII in a defocus range of 3-5 μm and 2232 micrographs of PEG-5000-ANSII in a range of 1-3 μm were taken.
Data processing - All image processing was performed using RELION-2.1-beta-0 [1]. Within RELION, movies were imported and movie alignment was performed with MotionCor2 [2] and subsequent CTF estimation was done with CTFFIND4 [3]. After CTF estimation, 147 micrographs of ANSII sample were selected. Manual picking of 235 particles allowed for generation of 3 initial 2D classes, subsequently used for automatically pick the whole dataset. In total, 211,651 particles were picked and extracted (144 pixel boxes) and subjected to 25 iterations of 2D classification. From the 100 generated classes, 19 are shown in Figure S2 representing 45,186 particles. For PEG5000-ANSII, 1828 micrographs were selected, 2650 particles manually picked to 3 initial 2D classes subsequently used for autopicking the whole dataset. 1,712,629 particles were automatically picked and subsequently classified in two rounds of 25 iterations. The 21 classes shown in Figure S1 represent 163,104 particles.

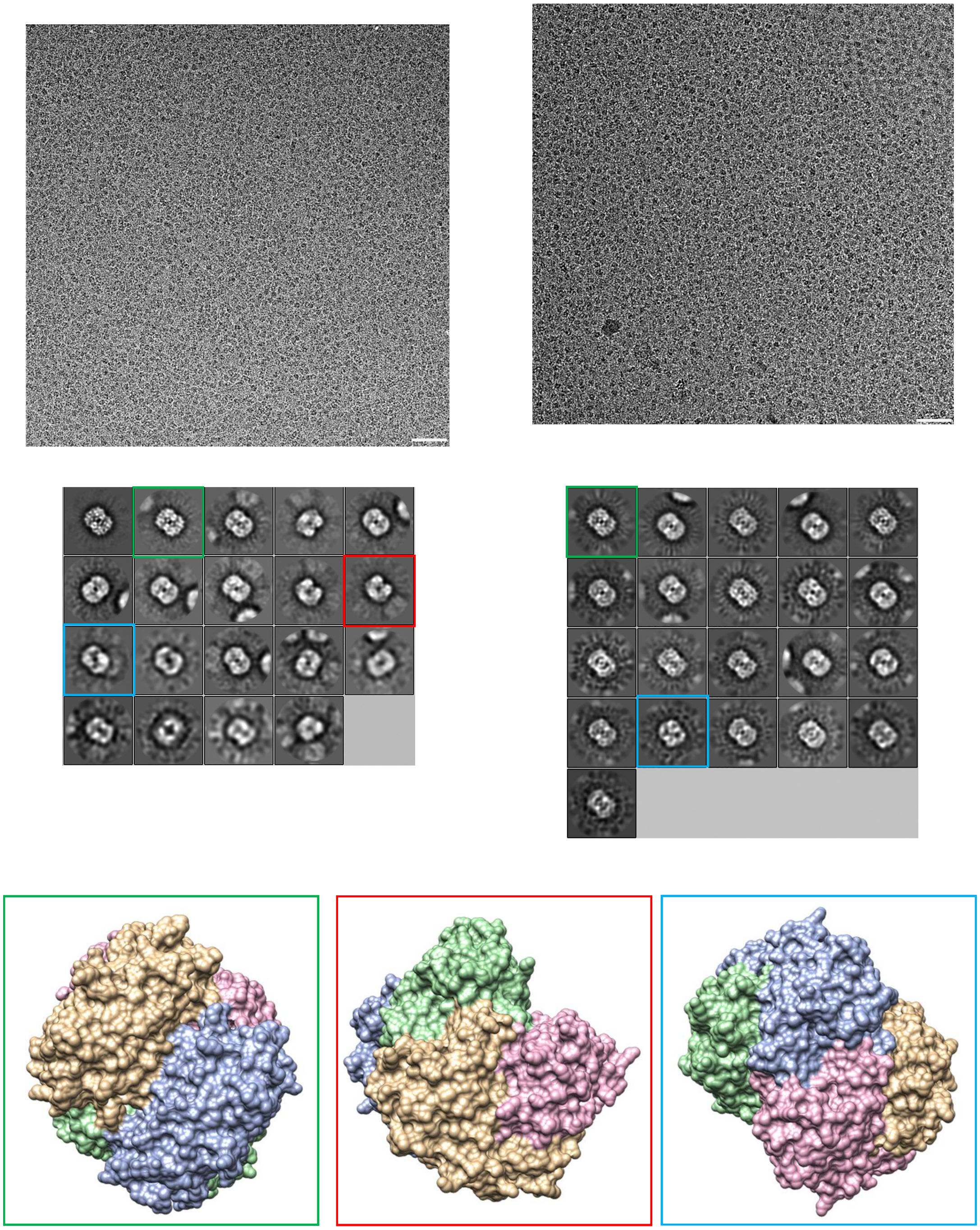

Figure S1. Cryo-EM micrograph and 2D classes of free ANSII (right) and ANSII PEGylated with PEG 5,000 (left). Micrographs scale bar is 50 nm. In the bottom, surface representation of ANSII (PDB: 3ECA) in three different orientations corresponding to the 2D classes.

Table S1. Tensor parameters of the RDCs collected in the presence of filamentous phages Pf1 to the calculated structural models with the lowest haddock-scores. The models were calculated either implementing only experimental SSNMR restraints or experimental SSNMR restraints together with RDCs. The tensor values were obtained for the tetrameric structures of ANSII using FANTEN user interface implemented in the WeNMR Portal [4]. The error of the Tensor parameters were evaluated using the Montecarlo Statistics in the program FANTEN.

Pf1 RDC Tensor	D^{HN} (Hz)	R	Q factor
Tetramer obtained by SSNMR restraints	−25 (± 1%)	−0.2 (± 3%)	0.29
Tetramer obtained by SSNMR restraints and RDCs	−21 (± 1%)	−0.2 (± 2%)	0.14

Table S2. HADDOCK statistics evaluated on the 200 water refined models of ANSII implementing either only experimental SSNMR restraints or experimental SSNMR restraints together with RDCs. The reported data are related to the best four structural models of the two main clusters with the lowest HADDOCK-scores.

	**Clust1 (SSNMR restraints)**	**Clust1 (SSNMR restraints and RDCs)**
**HADDOCK-Score**	−1228 ± 35	−1357 ± 34
**Nstruc**	66	178
**RMSD**	0.57 ± 0.34	0.43 ± 0.26
**Edesolv**	47 ± 10	35 ± 22
**BSA**	16974 ± 218	17103 ± 233
**Eair**	105 ± 29	89 ± 3

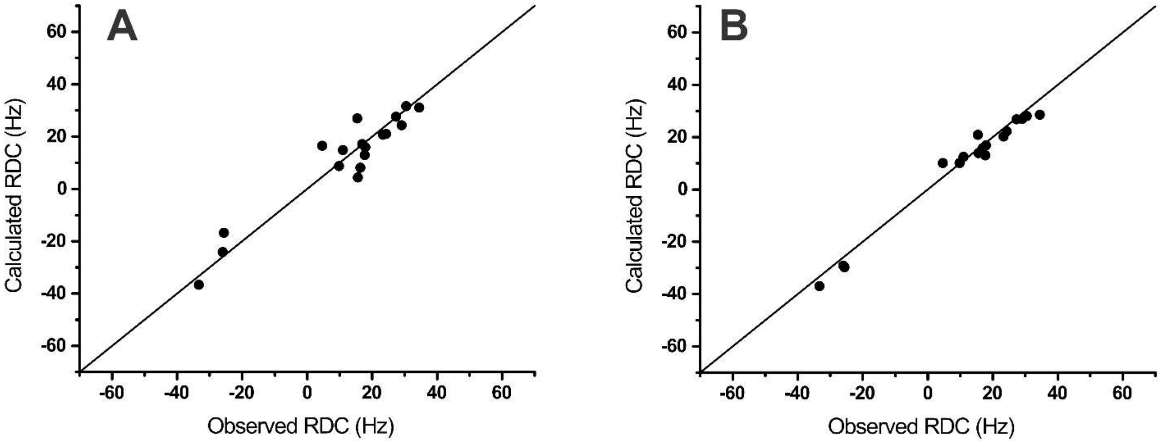

Figure S2. Fit of the experimental RDC values collected in the presence of filamentous phages Pf1 (used as alignment medium) to the structural model with the lowest haddock-score calculated implementing either (**A**) only experimental SSNMR restraints (Qfactor 0.29), or (**B**) experimental SSNMR restraints together with RDCs (Qfactor 0.14) in HADDOCK 2.2

1. Kimanius, D.; O. Forsberg, B.; Scheres, S.H.; Lindahl, E. Accelerated cryo-EM structure determination with parallelisation using GPUs in RELION-2. *eLife* **2016**, *5*.
2. Li, X.; Mooney, P.; Zheng, S.; Booth, C.; Braunfeld, M.B.; Gubbens, S.; Agard, D.A.; Cheng, Y. Electron counting and beam-induced motion correction enable near atomic resolution single particle cryoEM. *Nat. Methods* **2013**, *10*, 584–590.
3. Rohou, A.; Grigorieff, N. CTFFIND4: Fast and accurate defocus estimation from electron micrographs. *J. Struct. Boil.* **2015**, *192*, 216–221.
4. Linser, R.; Sarkar, R.; Krushelnitzky, A.; Mainz, A.; Reif, B. Dynamics in the solid-state: Perspectives for the investigation of amyloid aggregates, membrane proteins and soluble protein complexes. *J. Biomol. NMR* **2014**, *59*, 1–14.
